# Gut Microbiota Regulates Mincle Mediated Activation of Lung Dendritic Cells to Protect Against *Mycobacterium tuberculosis*

**DOI:** 10.3389/fimmu.2019.01142

**Published:** 2019-05-28

**Authors:** Shikha Negi, Susanta Pahari, Hilal Bashir, Javed N. Agrewala

**Affiliations:** ^1^Immunology Division, CSIR-Institute of Microbial Technology, Chandigarh, India; ^2^Immunology Division, Texas Biomedical Research Institute, San Antonio, TX, United States; ^3^Center for Biomedical Engineering, Indian Institute of Technology, Rupnagar, India

**Keywords:** gut-lung axis, antibiotics, mincle, tuberculosis, lung dendritic cells, T cells

## Abstract

Gut microbial components serve as ligand for various pattern recognition receptors (PRRs) present on immune cells and thereby regulates host immunity. Dendritic cells (DCs) are highly specialized innate cells involved in immune response to *Mycobacterium tuberculosis* (*Mtb*) infection. The gut-lung axis is a potential therapeutic target in tuberculosis; however, understanding of the innate immune mechanism underlying the interaction of gut microbiota and lung still remains obscure. We investigated if antibiotics (Abx) induced gut dysbiosis is able to affect the activation of innate receptor, macrophage inducible C-type lectin (mincle) in lungs during *Mtb* infection. We found that dysbiosis reduced the lung mincle expression with a concomitant increase in *Mtb* survival. Further, Abx diminished the effector and memory T cell population, while elevating frequency of regulatory T cells (Tregs) in the lungs. Here, we show that dysbiotic mice exhibited low mincle expression on lung DCs. These DCs with impaired phenotype and functions had reduced ability to activate naïve CD4 T cells, and thus unable to restrict *Mtb* survival. *In vivo* administration of trehalose-6,6-dibehenate (TDB: mincle ligand) efficiently rescued this immune defect by enhancing lung DCs function and subsequent T cell response. Further, gut microbial profiling revealed augmentation of *Lactobacillus* upon mincle stimulation in microbiota depleted animals. Accordingly, supplementation with *Lactobacillus* restored mincle expression on lung DCs along with anti-*Mtb* response. Our data demonstrate that gut microbiota is crucial to maintain DC-dependent lung immune response against *Mtb*, mediated by mincle. Abx interrupt this process to induce impaired T cell-response and increased susceptibility to *Mtb*.

## Introduction

Tuberculosis (TB) is the leading killer infectious disease caused by *Mycobacterium tuberculosis* (*Mtb*). Various *Mtb* and host factors decide the pathogenesis and outcome of the disease. Host gut microbiota, or signals derived from them can be an important determinant of lung immune response in *Mtb* infection. Moreover, recent evidence has reported changes in the gut microbial composition on *Mtb* exposure (1). Previously, we have also shown enhanced susceptibility to TB upon gut microbiota alteration (2).

Gut microbiota calibrates the host immunity by the array of mechanisms such as influencing the release of proinflammatory (IFN-γ, IL-17, IL-6, and IL-12) and anti-inflammatory IL-10 cytokines (3), metabolites release (4) and controlling function of mononuclear phagocytes including dendritic cells (DCs) (5, 6). Recent studies show that disturbance in gut microbiota is associated with the onset and progression of many diseases such as inflammatory bowel disease, autoimmunity, obesity and cancer (7–10). Thus, in addition to its local effects, gut microbiota modulates host immune response at extra-intestinal sites such as the brain, bone marrow and lung (5, 11–15).

Pattern recognition receptors (PRRs) expressed on immune cells are essential for the recognition of stimuli and further initiation of innate and adaptive immune response to infections. Microbial ligands derived from the intestine bind and activate PRRs; thus help in maintaining the homeostasis and immunity (16, 17). Moreover, stimulation of systemic immunity through translocation of gut microbial products from luminal side of the intestine into the blood circulation has been reported earlier (18). However, the role of intestinal microbes in regulating the function of PRRs in lung remains poorly understood in the case of TB.

Mincle (macrophage inducible C-type lectin) also known as Clec4e (C-type lectin domain family 4 member E) or Clecsf9 (C-type lectin superfamily member 9) is a PRR, involved in glycolipids recognition. It is expressed on immune cells such as DCs, macrophages, B cells and neutrophils (19). Mincle is known to sense various stimuli such as glycolipids derived from fungi (20), mycobacteria and other bacterial groups (21). Interestingly, gut commensal *Lactobacillus (L.) plantarum* derived cyclopropane-fatty acid α-glucosyl diglyceride (glycolipid) has been reported to bind and activate mincle (22). Further, trehalose-dibehenate (TDB) is a synthetic glycolipid analog known to signal through mincle (23). Various studies suggest the importance of mincle expression and activation in the immune response against *Mtb* (24, 25).

DCs have been known to efficiently present antigens to naïve CD4 T cells and elicit T cell polarization (26). Further, microbial ligands recognition by PRRs present on DCs modulates their function against various pathogens (27). Despite these findings, gut microbiota mediated regulation of *Mtb* survival through modulation of mincle receptor on lung DCs is currently unknown.

Herein, we show that dysbiosis of gut microbiota abrogated the mincle expression in lungs, thus compromising DCs function and subsequent T cell response against *Mtb*. These immune defects in the lung after Abx treatment could be restored by supplementation with TDB and *Lactobacillus*. These findings provide possible insights into the gut-lung crosstalk via innate receptors such as mincle expressed on lung DCs during *Mtb* infection.

## Materials and Methods

### Animals and Ethical Statement

Female C57BL/6 mice, 6–8 wk old were procured from animal house of the CSIR-Institute of Microbial Technology (IMTECH), Chandigarh, India. All experiments and protocols were in accordance with the Institutional Animal Ethics Committee of IMTECH and accredited by Committee for the Purpose of Control and Supervision of Experiments on Animals (No. 55/1999/ CPCSEA), Govt. of India.

### Reagents and Antibodies

All antibiotics, standard chemicals, and reagents used in the study were purchased from Sigma-Aldrich (St. Louis, MO, USA). Cytokines and fluorochrome-tagged antibodies were from BD Biosciences (San Diego, CA, USA) unless otherwise stated.

### Gut Microbiota Depletion in Mice by Abx Treatment

Mice were given broad-spectrum Abx cocktail (vancomycin, 0.5 g/l; neomycin sulfate, 1 g/l; and metronidazole, 1 g/l) *ad libitum* in drinking water for 4 wk to disrupt the gut microbiota. Abx containing water was changed twice a week and continued throughout the experiment. To check the load of cultivable microbes, serial dilutions of fecal samples were plated on BHI medium under aerobic and anaerobic conditions.

### Bacterial Strains Used in the Experiments

*Mtb* H37Rv was cultured to mid-log phase in Middlebrook 7H9 broth supplemented with 0.05% Tween-80, 0.2% glycerol and 10% OADC (Oleic Albumin Dextrose Catalase). Glycerol stocks of bacteria were stored at −80°C. The bacterial viability was determined by plating dilutions of bacteria on 7H11 agar plates through colony forming unit (CFU) assay. Colonies were enumerated after 21 days.

The bacterial strain *Lactobacillus plantarum* MTCC 2621 was acquired from the Microbial Type Culture Collection (MTCC), IMTECH. The culture was maintained on de Man Rogosa Sharp broth (Merck, Darmstadt, Germany) at 37°C, 5% CO_2_. Bacteria harvest was done by centrifugation at 1,250 × g for 10 min, and thereafter, washed twice followed by resuspension in PBS adjusting to 1 × 10^9^ CFU/ml. 1 × 10^8^ CFUs of bacteria were administered to mice through oral gavage every alternate day for 2 wk prior to *Mtb* infection until mice were sacrificed.

### Mouse Model of *Mtb* Infection

Mice were infected with H37Rv (~100 CFU) by aerosol challenge using inhalation exposure unit (Glas-Col, Terre Haute, IN, USA). The frozen stocks of the bacterium were thawed; washed twice with PBS and made into a single cell suspension by passing through insulin syringe. Animals were kept in BSL3 laboratory. *Mtb* burden was assessed in lung tissue homogenate by plating on 7H11 agar plates. CFUs were enumerated after 21 d of incubation at 37°C.

### Oral Administration of TDB

Trehalose-6,6-dibehenate (TDB) from InvivoGen (San Diego, CA, USA) was administered through oral gavage at a dose of 50 μg per mice 48 h prior to and after *Mtb* challenge.

### Isolation of Lung Cells

The lungs of mice were perfused with 10 ml of chilled PBS containing heparin (100 U/ml), whereupon lungs were removed and minced tissue were digested in collagenase (1 mg/ml) containing 20 μL/mL DNase I for 45–60 min at 37°C. Thereafter, cells were passed through a 70-μm cell strainer to prepare single cell suspension. Further, RBCs were removed using ACK lysis buffer (0.15 M NH_4_Cl, 10 mM KHCO_3_, 0.1 mM EDTA). The cells were washed with PBS and resuspended in complete medium (RPMI 1640/10% FBS).

### Quantitative Real-Time PCR (qRT-PCR)

Total RNA was isolated from lung tissue using TRIzol reagent (Invitrogen, Carisbad, CA, USA). The isolated RNA (1 μg) was used to synthesize cDNA with reverse transcription kit, according to the manufacturer's instructions (Applied Biosystems, Foster City, CA, USA). qRT-PCR was performed using SYBR green PCR mix (Applied Biosystems) according to manufacturer's protocol. The final PCR reaction was made in a volume of 10 μl, containing 0.2 μM forward primer, 0.2 μM reverse primer, SYBR green, and one-tenth of resulting cDNA was used as a template for PCR. Amplification was performed at 50°C for 2 min and 95°C for 10 min followed by 40 cycles of 95°C for 15 s and 60°C for 1 min in step one plus PCR (Applied Biosystems). Quantification of gene expression was depicted as fold change normalized to β-actin/and Glyceraldehyde 3-phosphate dehydrogenase (GAPDH) as an internal control (reference genes). Primers used in PCR are listed in Supplementary Table 1.

### Cytokines Quantification

Cytokines such as IFN-γ, IL-17, IL-10, IL-6, and IL-12 were measured in culture supernatants (SNs) by sandwich enzyme-linked immunosorbent assay (ELISA). Briefly, ELISA plates were coated overnight at 4°C with purified rat anti-mouse IFN-γ, IL-17, IL-6, IL-12 (2 μg/ml), and IL-10 (4 μg/ml) antibodies in phosphate buffer (pH 9.6, 0.05 M). The blocking of unbound sites was performed with BSA (1%) in PBS for 2–3 h at RT. Later, culture SNs (50 μl) and respective recombinant cytokines as standards were added to the wells and incubated overnight at 4°C. Later, biotin-labeled anti-mouse IFN-γ, IL-17, IL-6, IL-12, IL-10 (2 μg/ml) antibodies were added to the plates for 2 h at RT. Thereafter, avidin-HRP (1:10,000) was added and plates were incubated at 37°C for 1 h. The color was developed using H_2_O_2_-OPD. After color development, the reaction was stopped by H_2_SO_4_ (7%) and the plate was read at 492 nm. The cytokines amount was determined using the standard curve of recombinant cytokines log_2_ serial dilutions.

### Flow Cytometry

Cells were incubated with Fc block (anti-mouse CD16/32 antibody) at 4°C/30 min, to block the non-specific binding of antibodies to Fc receptors. This was followed by incubation of cells with fluorochrome tagged monoclonal antibodies (BD Biosciences, San Jose, CA) such as anti-CD4, anti-CD11c, anti-CD11b, anti-CD103, anti-F4/80, anti-CD44, anti-CD62L, anti-CD127, anti-CCR7, anti-Mincle, anti-CD86, anti-MHCII, and anti-PD1 along with their respective isotype matched control antibodies at 4°C/30 min. For staining intracellular marker such as FoxP3, cells were restimulated with phorbol 12-myristate 13-acetate (PMA, 50 ng/ml) and ionomycin (1 μg/ml) for 3 h followed by incubation of cells with brefeldin A (5 mg/ml) for an additional 2 h. Thereafter, cells were stained with anti- FoxP3 antibody at 4°C for 30 min. Samples were analyzed using BD FACS-Aria II and BD FACS DIVA software (BD Biosciences). The gating strategy used to analyse the T cells and DCs is represented in Figures S3, S4B.

### Proliferation Assay

CD4 T cells were incubated with carboxyfluorescein succinimidyl ester (CFSE, 2 μM) in PBS for 8 min at 37°C. Unbound CFSE was removed by washing cells three times with RPMI/10% FCS. Further, cells were cultured with PPD (25 μg/ml) for 72 h at 37°C/ 5% CO_2_. The proliferation of CFSE-labeled cells was assessed through flow cytometry.

### Lung DCs Isolation

CD11c^+^ cells were isolated from single cell lung suspension using CD11c^+^ magnetic beads (Miltenyi Biotec, Auburn, CA, USA) according to manufacturer's instructions. Briefly, cells were washed in 1 ml of MACS buffer, incubated on ice with CD11c^+^ beads for 30 min. Thereafter, cell suspension was washed twice with MACS buffer and passed through a magnetic column. The CD11c^+^ cells were then collected by positive selection. Macrophage population was removed from the CD11c^+^ cells by overnight plastic adherence incubation. Later, cells were washed with RPMI and enriched for DCs by culturing for 7 days in complete medium in presence of GM-CSF (20 ng; Pepro Tech, Rocky Hill, NJ, USA). Cultures were fed every 48 h. The purity of DCs was >90% as enumerated by flow cytometry.

### Isolation of CD4 T Cells and Coculture With DCs

CD4 T cells were enriched from lungs of *Mtb* challenged mice using CD4 T cell enrichment kit (BD Biosciences). Briefly, single cell suspension of lung was made followed by ACK lysis to remove RBCs. Later, CD4 T cells were isolated using BD IMag™ mouse CD4 T lymphocyte enrichment set–DM (BD Biosciences) through MACS by negative selection. The purity of isolated CD4 T cells was >95% as monitored by flow cytometry.

Thereafter, DCs were cocultured with CFSE labeled CD4 T cells in a ratio of 1:10 with PPD (25 μg/ml) at 37°C/5% CO_2_. After 72 h, proliferation of CD4 T cells was assessed by flow cytometry. The cultures SNs were collected to estimate cytokines by ELISA.

### *Mtb* Uptake Assay by DCs

DCs (2.5 × 10^5^/well) were infected with H37Rv at 5 multiplicity of infection (MOI) for 4 h at 37°C/5% CO_2_. Thereafter, extracellular *Mtb* was removed by treatment with amikacin (2 μg/ml). It was followed by lysis of cells with saponin and plating on 7H11 agar plates. The colonies were counted 3 wk after the incubation of plates at 37°C/5% CO_2_.

### Adoptive Transfer of DCs

Purified lung DCs from *Mtb* infected mice were PPD-pulsed and stimulated or unstimulated with TDB (20 μg/ml) prior to injecting intravenously (i.v.) into the normal and Abx treated mice at the amount of 5 × 10^6^ cells/mice in 200 μl sterile PBS.

### Gut Microbiota Analysis

Fecal DNA was isolated using DNA extraction kit (Zymo Research, Irvine, CA, USA). Further, DNA was amplified from the V3-V4 region of 16S rRNA gene and sequencing was performed using Illumina MiSeq. Sequences were processed in the QIIME pipeline (28). Further, sequences were split and trimmed to a minimum quality score of 20, aligned to the reference Greengenes database using UCLUST (29) and a reference OTU threshold of 97%. The analysis was done for abundance at phylum and genus level. The samples were normalized using ‘cumulative sum scaling' (CSS) (30).

For qPCR analysis, 50–100 ng of isolated DNA was used in PCR reaction with SYBR green PCR mix (Applied Biosystems, Foster City, CA, USA) according to manufacturer's protocol. Briefly, PCR reaction was performed in a volume of 10 μl, containing 0.2 μM forward primer, 0.2 μM reverse primer, SYBR green, and fecal DNA as template. qPCR amplification was performed at 50°C for 2 min and 95°C for 10 min followed by 40 cycles of 95°C for 15s and 60°C for 1 min in step one plus PCR (Thermo Fisher Scientific, Waltham, MA, USA). Relative abundance of bacteria was depicted as fold change and normalized with universal bacteria control. Primers used in PCR are listed in Supplementary Table 1.

### Statistical Analysis

Data analysis was done using one-way ANOVA for multiple group comparisons; unpaired Student's *t*-test was used for two group comparisons with GraphPad Prism 5 (GraphPad, San Diego, CA, USA). *p* < 0.05 was considered as statistically significant.

## Results

### Abx Treatment Reduces the Expression of Mincle in Lung and Promotes *Mtb* Survival

**Figure 1:**
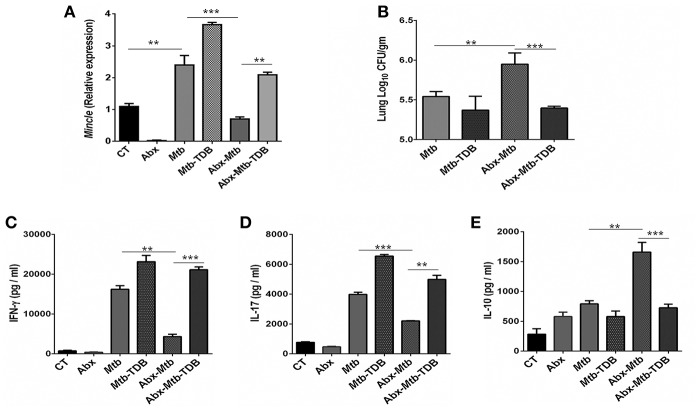
Downregulation of mincle receptor in lungs of gut microbiota disrupted animals dampens anti-*Mtb* immunity. Mice were given *ad libitum* access to Abx supplemented drinking water for 4 wk, prior to aerosol challenge with *Mtb* (~100 CFU) and oral administration of TDB (50 μg). At 30 d post infection, lung cells were harvested and assessed for the **(A)** expression of mincle by qRT-PCR, depicted as fold change relative to control and normalized to β-actin and GAPDH reference gene; **(B)** *Mtb* burden by CFU assay. Further, **(C–E)** lung lymphocytes were stimulated with PPD (25 μg/ml) for 48 h. Thereafter, culture SNs was collected and quantified by ELISA for the secretion of **(C)** IFN-γ, **(D)** IL-17, and **(E)** IL-10. Data represented as mean ± SD are of three independent experiments (*n* = 5 mice/group). ^**^p < 0.01, ^***^p < 0.001. CT: control mice without Abx treatment; Abx: mice treated with Abx; *Mtb*: *Mtb* challenged mice; *Mtb*-TDB: mice with *Mtb* infection and TDB administration; Abx-*Mtb*: mice treated with Abx prior to *Mtb* infection; Abx-*Mtb*-TDB: mice with disrupted gut microbiota prior to *Mtb* infection and TDB administration.

To investigate the effect of gut dysbiosis on mincle receptor and thereby upon lung immunity, mice were treated with broad-spectrum Abx prior to *Mtb* infection and TDB administration. Abx treatment resulted in a considerable reduction of commensal aerobic (*p* < 0.001) and anaerobic (*p* < 0.001) bacteria (Figures S1A,B). As shown in Figure 1A, *Mtb* infection induced the expression of mincle receptor in the lungs. However, in comparison to *Mtb* challenged mice, Abx significantly reduced the mincle level in Abx-*Mtb* group. Additionally, other innate receptors such as TLR-2, NOD-2 and Dectin-1 did not show any significant changes upon Abx treatment (Figure S2). Further, oral administration of TDB resulted in a substantial upregulation of mincle (*p* < 0.01) in the Abx-*Mtb*-TDB group (Figure 1A). Consistent with this observation, we found the considerable reduction (*p* < 0.001) of *Mtb* burden in Abx-*Mtb*-TDB mice compared with Abx-*Mtb* (Figure 1B).

Proinflammatory cytokines such as IFN-γ and IL-17 contribute to the host protection against *Mtb* (31, 32). In contrast, IL-10 is known to exert suppressive immune response (33). Thus, we next assessed the level of these cytokines in the lungs of gut microbiota-disrupted mice after mincle stimulation. We observed restoration of IFN-γ (*p* < 0.001) and IL-17 (*p* < 0.01) in the Abx-*Mtb*-TDB group that was declined upon gut dysbiosis in Abx-*Mtb* mice (Figures 1C,D). Further, IL-10 was increased in Abx-*Mtb*, which was however decreased on TDB supplementation (Figure 1E). These data indicate that mincle stimulation in gut microbiota depleted mice effectively restricts *Mtb* survival and promotes the generation of protective proinflammatory response in the lung.

### Mincle Stimulation Inhibits the Frequency of Tregs and PD-1^+^ CD4 T Cells in Lungs of Mice With Gut Dysbiosis

**Figure 2:**
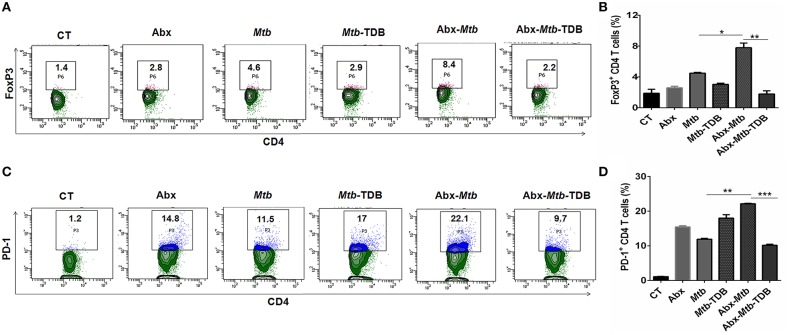
Mincle stimulation reduces Tregs population and frequency of PD-1^+^ CD4 T cells in gut microbiota disrupted animals. Mice were treated as described in the legend to Figure 1. Lymphocytes isolated from lungs were *in vitro* stimulated with PPD (25 μg/ml) for 72 h. Thereafter, expression of **(A,B)** intracellular FoxP3 and **(C,D)** PD-1 were assessed on CD4 T cells by flow cytometry. Contour plots and bar graphs represent the percentage of CD4 FoxP3^+^ and CD4 PD-1^+^ gated population. Data are shown as mean±SD of 2–3 independent experiments (*n* = 5 mice/group). ^*^p < 0.05, ^**^p < 0.01, ^***^p < 0.001.

Next, we were interested to examine the impact of mincle stimulation on the levels of *Mtb* specific Tregs in lungs of dysbiotic mice. Tregs are known to delay the accumulation of effector T cells in the *Mtb* infected lungs (34). We observed that as compared to Abx-*Mtb* group, signaling through mincle significantly reduced the percentage of *Mtb* specific FoxP3^+^ CD4 Tregs (*p* < 0.01) in Abx-*Mtb*-TDB mice (Figures 2A,B).

Further, *Mtb* provokes the induction of exhausted T cells that is another impediment in preserving immunity against *Mtb* owing to their impaired function (35). As compared to *Mtb* challenged mice, a high percentage of PD-1^+^ CD4 T cell was noted in lung of microbiota disrupted mice (Abx-*Mtb*). It is worth to mention here that administration of TDB sufficiently restored the functional unresponsiveness of CD4 T cells, as evident by the declined pool of PD-1^+^ CD4 T cells (*p* < 0.001) in Abx-*Mtb*-TDB group compared to Abx-*Mtb* mice (Figures 2C,D). These data suggest that intestinal cues regulate the suppressive Treg and exhausted T cell population in lung during *Mtb* infection via mincle.

### Triggering Through Mincle Expands the Activated and Memory CD4 T Cell Pool Against *Mtb* in Lungs of Mice With Disrupted Gut Microbiota

**Figure 3:**
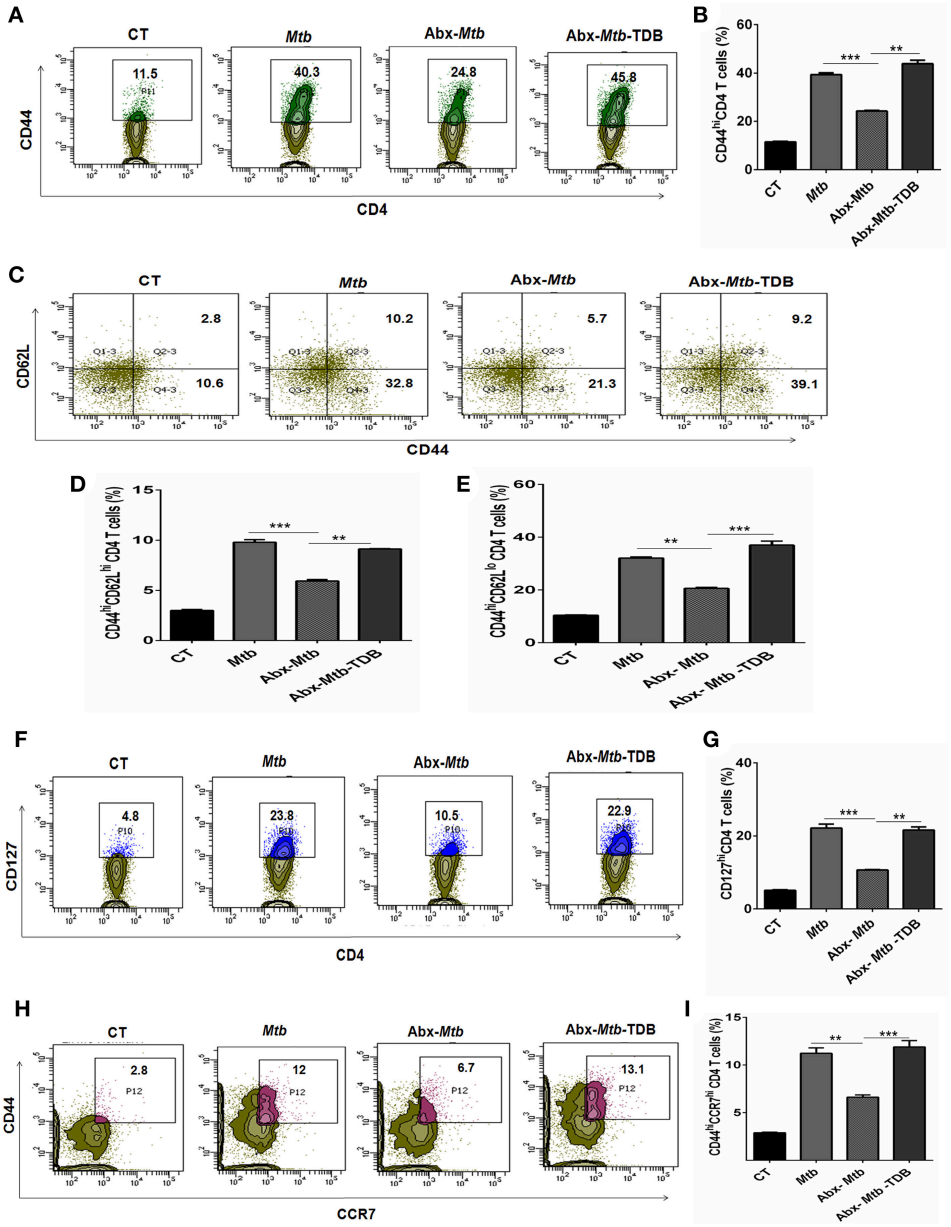
Triggering through mincle restores the percentage of effector and memory CD4 T cells in mice with gut dysbiosis. Gut microbiota-disrupted mice were given *Mtb* challenge and TDB supplementation as mentioned in the legend to Figure 1. After 4 wk, lung lymphocytes were isolated and stained *ex vivo* for the T cells activation and memory markers, thereafter assessed by flow cytometry. CD4 T cells gated population was monitored for the **(A,B)** CD44^hi^ expression representing activated CD4 T cells; **(C–E)** coexpression of CD44 and CD62L for effector (CD44^hi^ CD62L^lo^) and central (CD44^hi^ CD62L^hi^) memory CD4 T cells; **(F,G)** CD127^hi^; **(H,I)** CD44^hi^ CCR7^hi^ cells. The results depicted as contour plots and bar diagram indicate percent population of the gated cells. The data represented as mean ± SD of three independent experiments (*n* = 5 mice/group). ^**^p < 0.01, ^***^p < 0.001.

The hallmark of effective and prolonged immunity is the generation of immunological memory (36). The impact of gut microbiota in the differentiation and maintenance of lung memory T cells in response to *Mtb* infection is still unexplored. Intriguingly, the frequency of activated CD4 T cells (*p* < 0.001) was markedly diminished in Abx-*Mtb* mice in comparison to *Mtb* challenged group (Figures 3A,B). Furthermore, these mice exhibited lesser prevalence of central and effector memory T cells, as characterized by a decline in the pool of CD62L^hi^CD44^hi^ and CD62L^lo^CD44^hi^ expressing CD4 T cells, respectively.

Interestingly, as compared to Abx-*Mtb* mice, TDB administered animals (Abx-*Mtb*-TDB) showed significant restoration in the percentage of the activated (*p* < 0.01), central memory (*p* < 0.01) and effector memory (*p* < 0.001) CD4 T cells (Figures 3C–E). We substantiated our finding by examining the expression of CD127, a marker known to maintain the survival of memory T cells (37). We found a sizeable increment of CD127^hi^ CD4 T cells (*p* < 0.01) in the Abx-*Mtb*-TDB group, in comparison to Abx-*Mtb* (Figures 3F,G).

We also examined the expression of CCR7 marker, which is involved in the memory CD4 T cells migration (38). Interestingly, we observed a significant restoration of CCR7^hi^CD44^hi^ expressing CD4 T cells (*p* < 0.001) in the Abx-*Mtb*-TDB mice (Figures 3H,I). These results signify the activation and expansion in the pool of lung effector and memory CD4 T cells upon mincle signaling in the gut microbiota depleted animals.

### Gut Dysbiosis Impairs the Phenotype and Cytokine Response of Mincle Expressing Lung DCs

**Figure 4:**
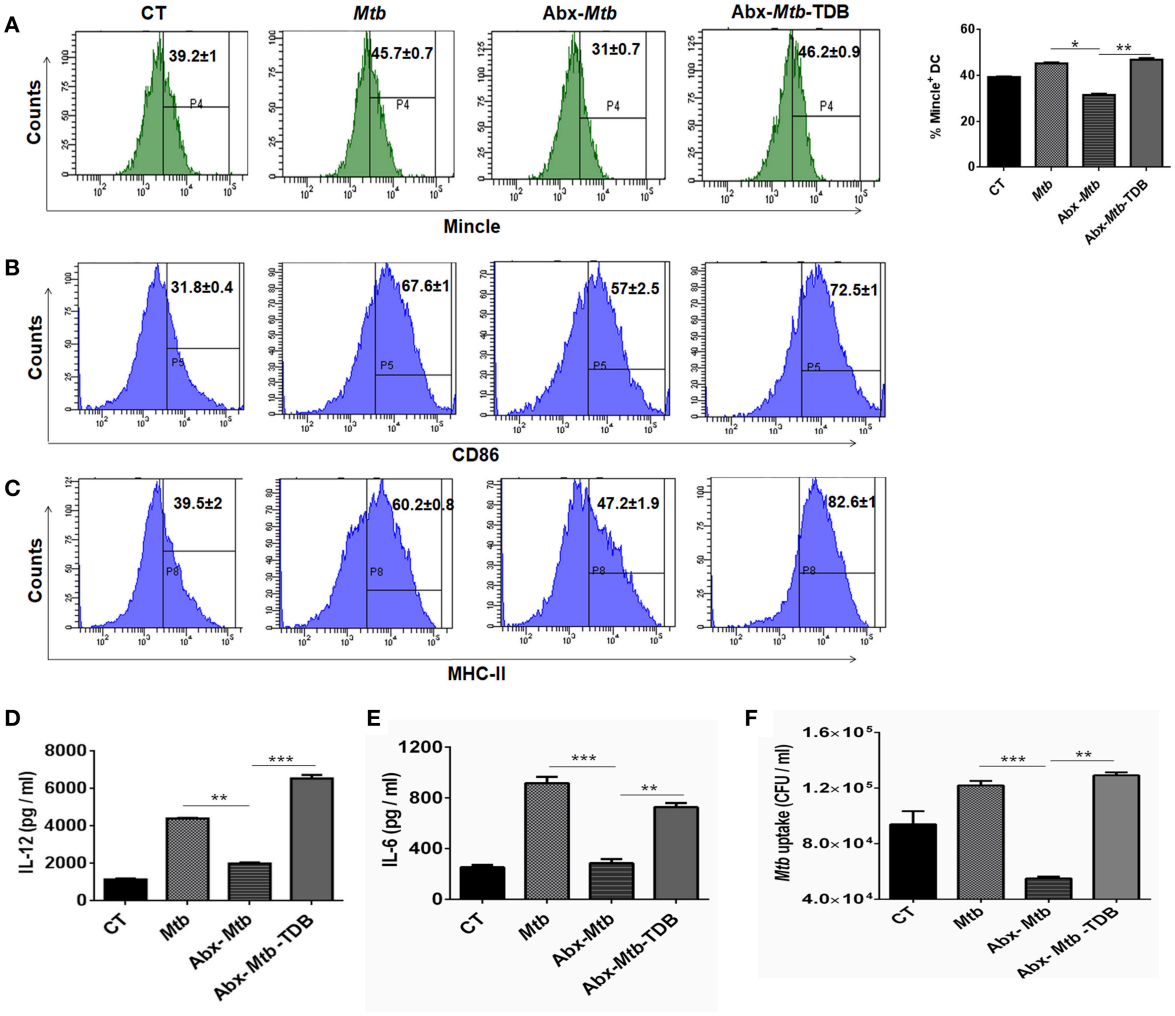
Stimulation through mincle reinstates the lung DCs function in gut microbiota depleted animals. *Mtb* infected mice with ablated gut microbiota were orally gavaged with TDB as indicated in legend to Figure 1. After 3 wk, lung cells from different groups of mice were sorted for CD11c^+^ population and **(A–C)** stained for the expression of **(A)** mincle receptor, bar graph depicts percentage of mincle positive population; **(B)** CD86; **(C)** MHC-II. Insets of flow cytometry represent percentage of cells gated on CD11c^+^ CD11b^+^ population. Further, **(D,E)** enriched lung DCs were pulsed with PPD (15 μg/ml) overnight. Later, cytokines **(D)** IL-12; **(E)** IL-6 were estimated in the cell culture SNs; **(F)** *Mtb* uptake by DCs was assessed through CFU assay. Data shown as means ± SD are of 2–3 independent experiments (*n* = 6 mice/group). ^*^p < 0.05,^**^p < 0.01, ^***^p < 0.001.

Next, we focused on innate cells, DCs as they can shape naïve CD4 T cell response by serving as efficient antigen-presenting cells and modulating the cytokine environment of T cell polarization (26). To accomplish this, lung cells were sorted for CD11c^+^ population and assessed for mincle expression. We noticed that as compared to *Mtb* infected group, lung DCs which are CD11c^+^ CD11b^+^ and not other subsets of cells (Figure S4A) exhibited lower expression of mincle in Abx-*Mtb* mice as examined by flow cytometry (Figure 4A) and further confirmed by qRT-PCR (Figure S5). This observation correlated well with the reduced activation and antigen presentation ability of lung DCs, as evident in the reduced expression of CD86 and MHC-II molecules respectively, (Figures 4B,C).

Further, the ability of lung DCs isolated from Abx-*Mtb* mice to secrete pro-inflammatory cytokines such as IL-12 (*p* < 0.01) and IL-6 (*p* < 0.001), was also impaired in comparison to DCs from *Mtb* challenged mice (Figures 4D,E). Additionally, we tested the phagocytic ability of these DCs that is an important attribute for effective antigen presentation to T cells (26). Consistent with the above findings; DCs from Abx-*Mtb* mice showed diminished *Mtb* uptake, as indicated by the lower number of bacterial CFUs (Figure 4F). Interestingly, administration of TDB in dysbiotic mice with *Mtb* infection upregulated the expression of the mincle receptor on lung DCs, restored their activated phenotype and cytokines production.

### Gut Dysbiosis Abrogates the Ability of Mincle Expressing Lung DCs to Promote Th1 and Th17 Immune Response

**Figure 5:**
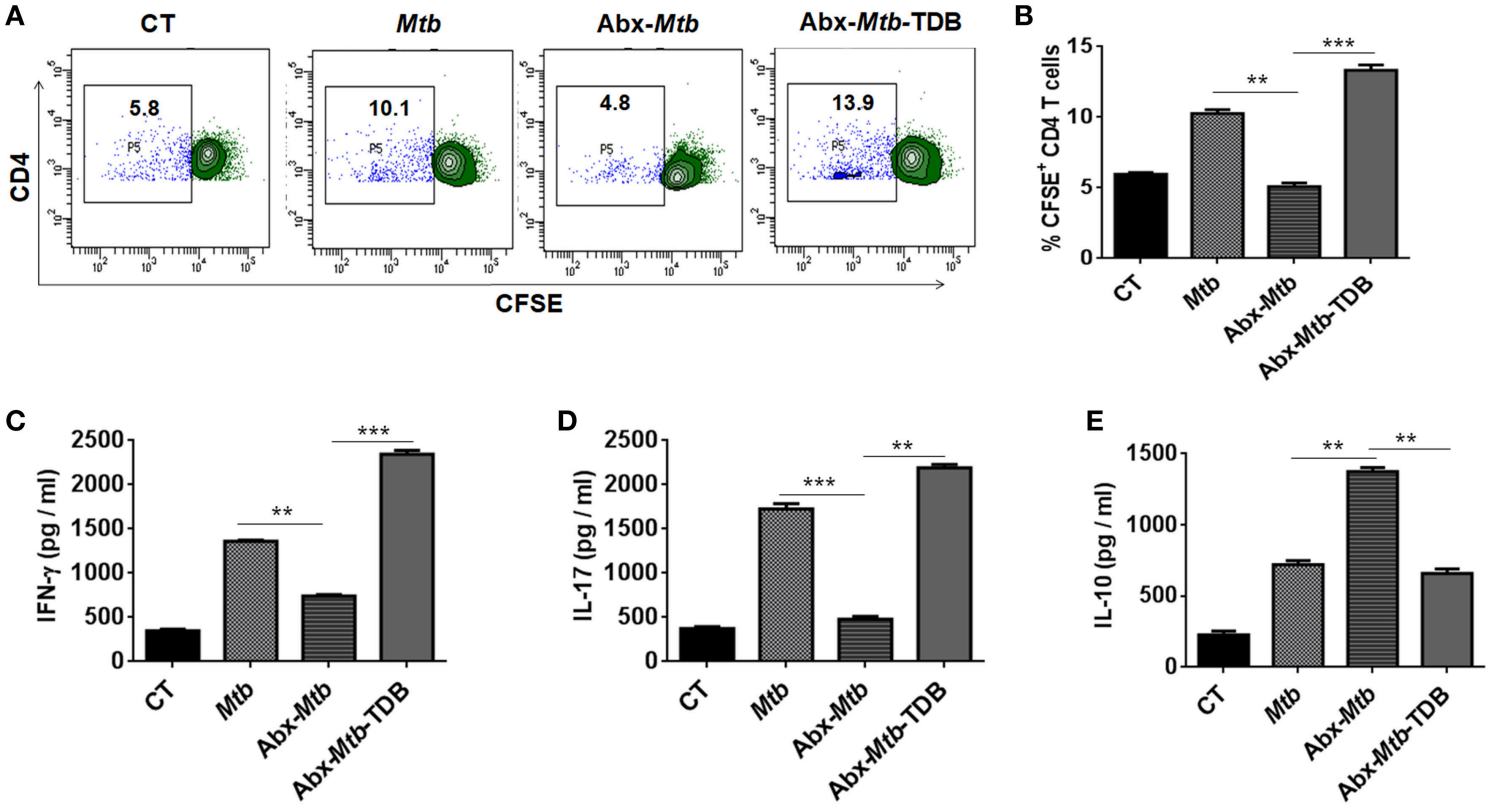
Lung DCs from gut microbiota ablated mice fail to stimulate *Mtb* specific naïve CD4 T cells. Mice treated with Abx were infected with *Mtb* and orally administered TDB as illustrated in the legend to Figure 1. After 3 wk, sorted CD11c^+^ lung cells enriched for DCs were pulsed with PPD (15 μg/ml) overnight prior to coculture with *Mtb* specific naïve CD4 T cells which were prior labeled with CFSE (2 μM) at a ratio of 1:10. After 72 h, proliferation of CD4 T cells was examined by flow cytometry. **(A)** Contour plots and **(B)** Bar graph represent the percent population of CFSE^+^ CD4 T cells. Later, **(C)** IFN-γ, **(D)** IL-17, and **(E)** IL-10 level was measured in the culture SNs by ELISA. Data are shown as mean ± SD of 2–3 independent experiments (*n* = 6 mice/group). ^**^p < 0.01, ^***^p < 0.001.

Further, we examined the ability of lung DCs to activate and differentiate CD4 T cells. Thus, lung DCs cells from different groups were cocultured with *Mtb*-specific CD4 T cells isolated from *Mtb* primed mice and *in vitro* pulsed with PPD for 72 h. DCs from Abx-*Mtb*-TDB group, compared to Abx-*Mtb* induced greater proliferation (*p* < 0.001) of CD4 T cells (Figures 5A,B). Moreover, we observed an elevated production of IFN-γ (*p* < 0.001) and IL-17 (*p* < 0.01) cytokines while there was decline in the level of IL-10 (*p* < 0.01) (Figures 5C–E). These findings indicate that lung DCs from Abx treated mice were defective in inducing Th1 and Th17 immune response, which was in accordance with the observed decrease in their MHC-II and CD86 expression.

### Abx Mediated Impairment in Lung DCs Function Involves Defective Mincle Activation

**Figure 6:**
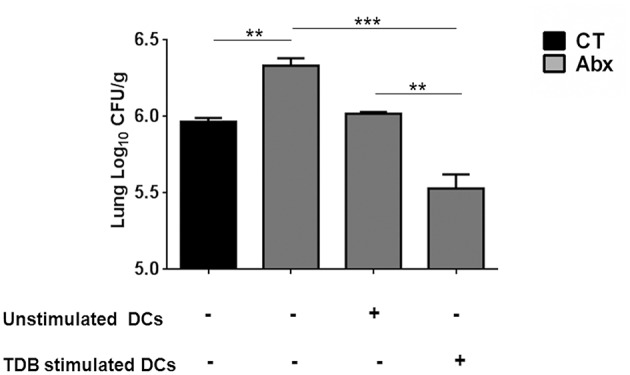
Adoptive transfer of mincle stimulated DCs restricts *Mtb* survival in Abx treated mice. Lung DCs (5 × 10^6^) from *Mtb* infected mice were PPD pulsed and stimulated with TDB (20 μg/ml) overnight. Cells were adoptively transferred to Abx treated mice, 24 h prior to *Mtb* infection. After 3 wk, *Mtb* burden was monitored in the lungs by CFU assay. Data represented as mean ± SD are of 2 independent experiments (*n* = 5 mice/group). ^**^p < 0.01, ^***^p < 0.001.

We wanted to ascertain the direct effect of mincle stimulation on lung DCs ability to restrict *Mtb* survival in dysbiotic mice. For this, PPD pulsed lung DCs (isolated from *Mtb* infected mice) either unstimulated or stimulated with TDB were adoptively transferred into mice with disrupted gut microbiota. Strikingly, we observed a significantly lesser *Mtb* burden (*p* < 0.01) in the lungs of mice that received mincle activated DCs, in comparison to the unstimulated DCs (Figure 6).

**Figure 7:**
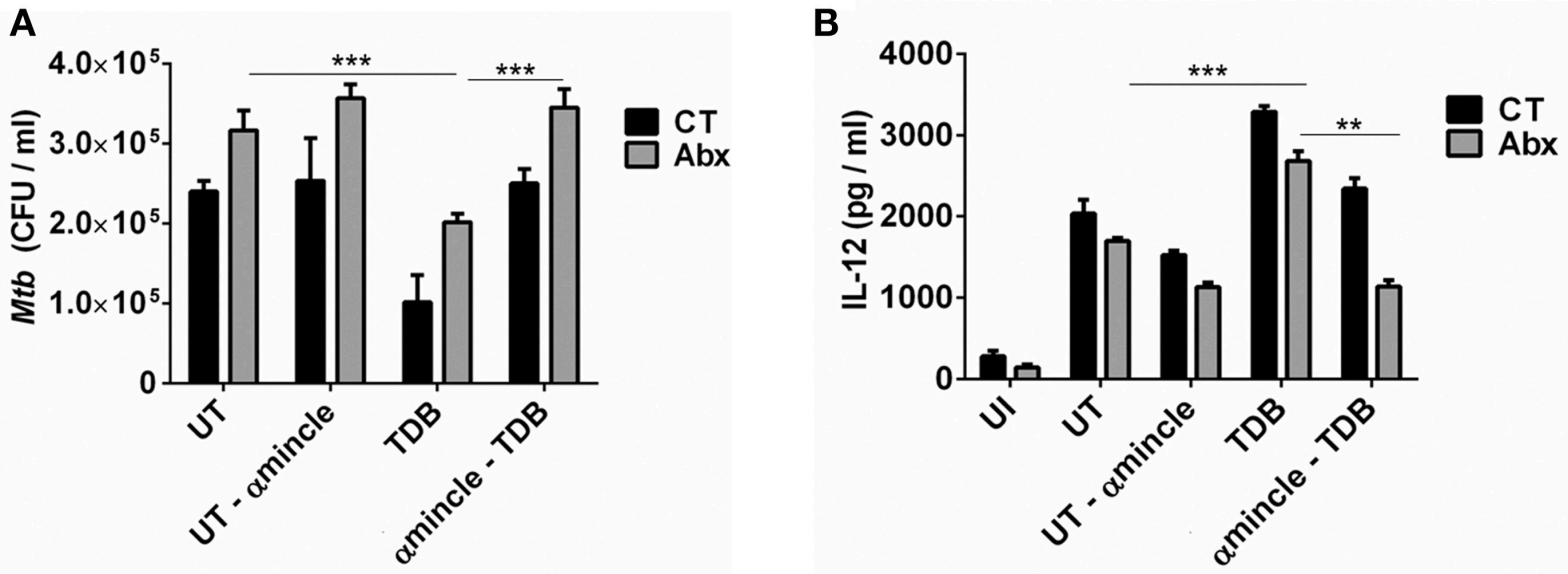
Blocking of mincle receptor in lung DCs limits their ability to restrict the growth of *Mtb*. Lung DCs from CT and Abx treated mice were infected with *Mtb* (at multiplicity of infection 5) *in vitro* and incubated with or without anti-mincle blocking antibody (α mincle; 10 μg/ml) for 1 h prior to TDB stimulation (20 μg/ml). After 48 h, **(A)** *Mtb* killing ability of DCs was examined by CFU assay; and **(B)** IL-12 secretion in the culture SNs quantified by ELISA. Data represented as mean ± SD are of 2 independent experiments (*n* = 5 mice/group). ^**^p < 0.01, ^***^p < 0.001., UI: uninfected DCs; UT: *Mtb* infected DCs; UT- α mincle: DCs infected with *Mtb* and treated with anti-mincle blocking antibody; TDB: *Mtb* infected DCs stimulated with TDB; TDB-α mincle: *Mtb* infected DCs treated with anti-mincle blocking antibody prior to TDB stimulation.

Next, to further clarify the role of mincle activation in lung DCs from dysbiotic mice, we inhibited the mincle receptor in *Mtb* infected DCs isolated from antibiotics treated mice with anti-mincle blocking antibody (α mincle). Interestingly, blocking mincle receptor resulted in impaired *Mtb* clearance by these DCs as evident in high CFU counts (Figure 7A). Additionally, these DCs failed to produce Th1 promoting cytokine such as IL-12 in response to *Mtb* infection (Figure 7B). Together, these results indicate that diminished mincle stimulation in DCs upon gut dysbiosis is the possible reason behind increased *Mtb* survival.

### Mincle Stimulation Shifts the Gut Microbial Composition in Abx Treated Mice Infected With *Mtb*

**Figure 8:**
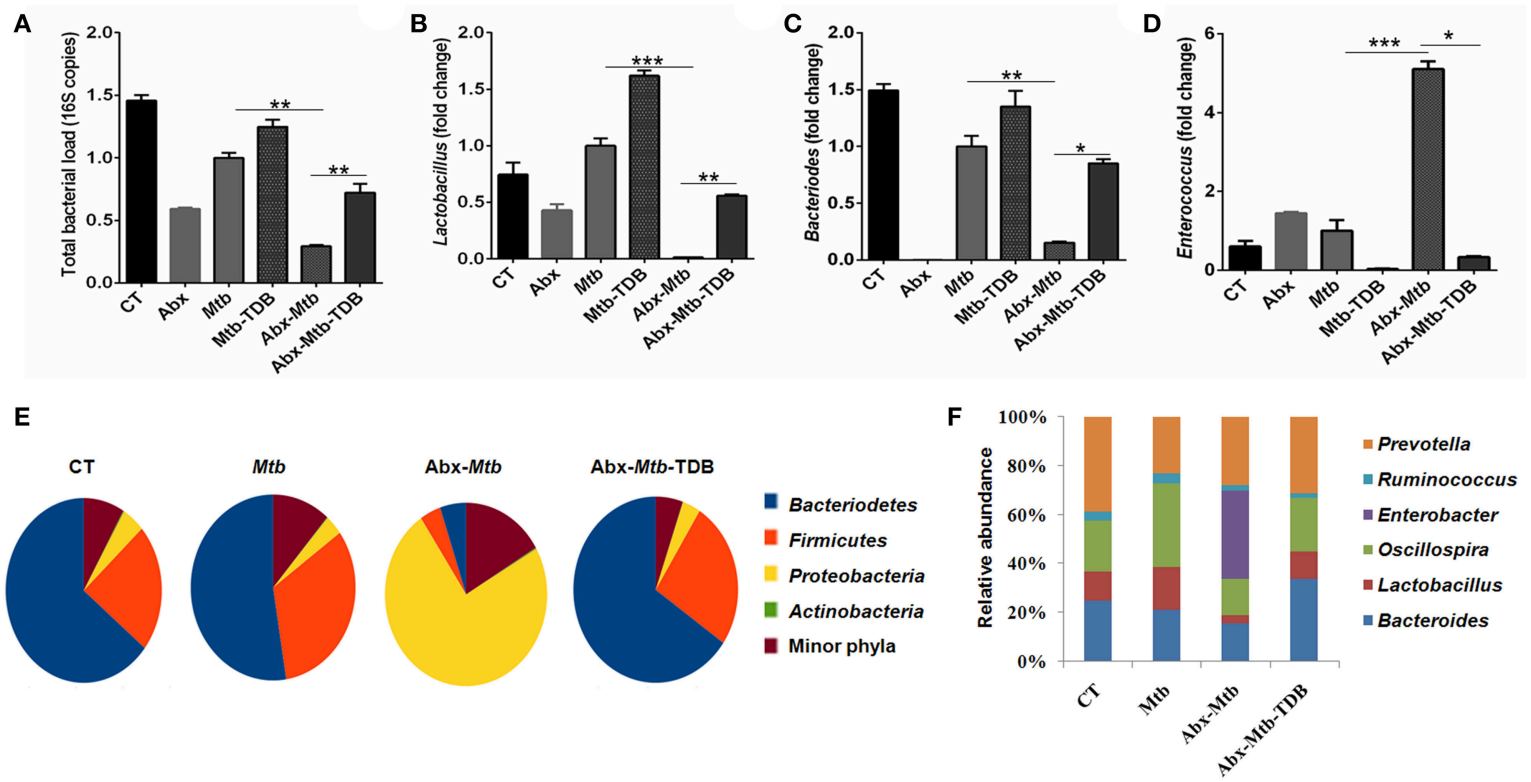
Triggering through mincle induces distinct shifts in the gut microbiota composition. Mice were treated with broad-spectrum Abx for 4 wk, followed by *Mtb* infection and TDB supplementation. After 30 d post infection, fecal DNA was isolated and subjected to **(A–D)** qPCR analysis, Bar graphs depict **(A)** total gut bacterial load; **(B)** *Lactobacillus*; **(C)** *Bacteroides*; **(D)** *Enterococcus*. Relative abundance depicted as fold change normalized with a universal bacterial primer, ^*^p < 0.05, ^**^p < 0.01, ^***^p < 0.001. Further, **(E,F)** 16S rRNA gene sequencing of fecal DNA was performed on Illumina MiSeq platform. **(E)** Relative abundance at phylum level was depicted in pie chart; **(F)** Bar diagram represents relative abundance of bacterial genera. Data are from two independent experiments (*n* = 5–6 mice/group).

Next, we investigated whether the observed restoration of the immune response in gut-disrupted mice on mincle activation is related to changes in gut microbial composition. Interestingly, qPCR analysis of fecal DNA revealed recovery of total bacterial load (*p* < 0.01) in Abx-*Mtb*-TDB mice as compared to Abx-*Mtb* (Figure 8A). Moreover, there was a significant abundance of *Lactobacillus* (*p* < 0.01) and *Bacteroides* (*p* < 0.05) while *Enterococcus* (*p* < 0.05) genus was considerably decreased in the Abx-*Mtb*-TDB mice relative to the Abx-*Mtb* group (Figures 8B–D).

Further, 16S rRNA sequencing showed that mincle activation noticeably increased the level of phylum *Bacteriodetes* and Firmicutes in the Abx-*Mtb*-TDB mice, which was found to be depleted in the Abx-*Mtb* group. In contrast, Proteobacteria was less abundant in these animals (Figure 8E). At the genus level, perceptible changes were seen in *Bacteroides, Oscillospira, Ruminococcus*, and *Lactobacillus*. Strikingly, mice in Abx-*Mtb*-TDB group relative to Abx-*Mtb* displayed an increase in *Lactobacillus* and *Bacteroides* commensals population (Figure 8F). These changes were in line with the qPCR data (Figures 8B,C).

### Administration of *Lactobacillus plantarum* Restores the Anti-*Mtb* Immunity in Gut Microbiota-Disrupted Mice

**Figure 9:**
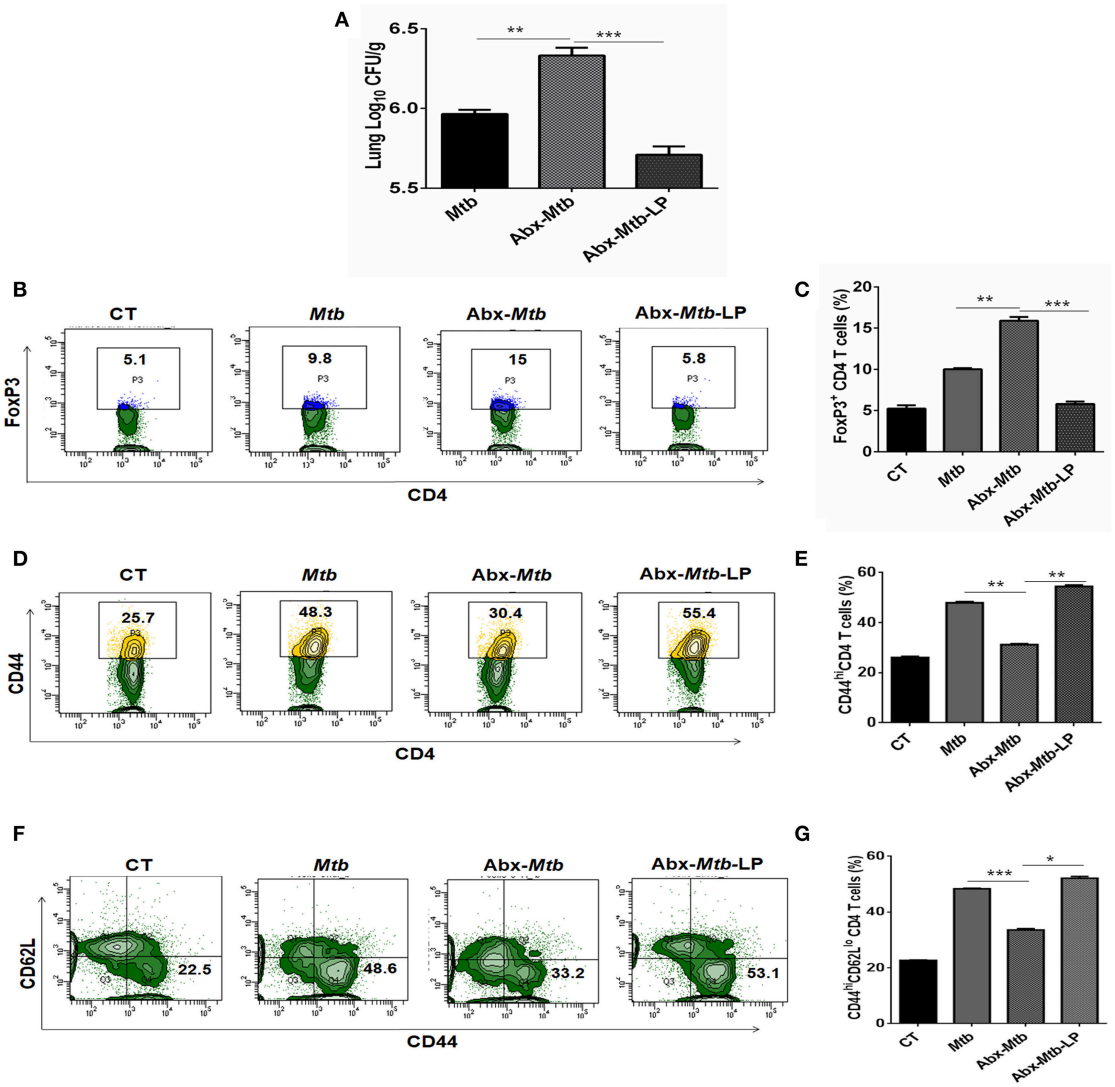
*Lactobacillus plantarum* restores the expression of mincle in lung along with compromised immunity against *Mtb* in mice with gut dysbiosis. Mice were subjected to Abx in drinking water for 4 wk followed by *L. plantarum* (10^8^ CFU per mice) administration every other day for 2 wk prior to *Mtb* infection until sacrifice. After 4 wk, lung tissue was harvested and examined for the **(A)** *Mtb* load by CFU assay. Further, **(B,C)** lung lymphocytes were cultured with PPD (25 μg/ml) for 72 h. Later, cells were evaluated for the expression of FoxP3 on CD4 T cells by flow cytometry. **(D–G)** Lung cells were monitored *ex vivo* for the expression of CD44 and CD62L to evaluate the **(D,E)** activation (CD44^hi^); and **(F,G)** effector memory response (CD62L^lo^CD44^hi^) by flow cytometry. Contour plots and bar graphs represent the percentage population of cells gated on CD4 T cells. Data represented as mean±SD are of 2 independent experiments (*n* = 5 mice/group). CT: control mice with no Abx; *Mtb*: *Mtb* challenged mice; Abx-*Mtb*: mice treated with Abx prior to *Mtb* infection; Abx-*Mtb*-LP: Abx treated mice infected with *Mtb* and supplemented with *Lactobacillus plantarum*. ^*^p < 0.05, ^**^p < 0.01, ^***^p < 0.001.

*Lactobacillus (L.) plantarum* derived glycolipids has been reported to signal through mincle receptor (22). In accordance to results in Figure 8B, we observed a lower abundance of *L. plantarum* (member of *Lactobacillus* genus) in Abx-*Mtb* group (Figure S6). Thus, we next examined the effect of *L. plantarum* on lung immunity against *Mtb* during gut dysbiosis. Oral administration of *L. plantarum* to microbiota depleted mice (Abx-*Mtb*-LP) upregulated the expression of mincle and MHC-II on lung DCs as compared to Abx-*Mtb* group (Figures S7A,B). This observation was concomitant with the reduction in lung *Mtb* burden (*p* < 0.001, Figure 9A) and suppressive Tregs population (Figures 9B,C). Additionally, there was an increase in the frequency of activated and effector memory CD4 T cells characterized by CD44^hi^ (Figures 9D,E) and CD62L^lo^CD44^hi^ (Figures 9F,G) expression, respectively. These data indicate that *L. plantarum* was able to enhance lung immunity against *Mtb* in dysbiotic mice.

## Discussion

**Figure 10:**
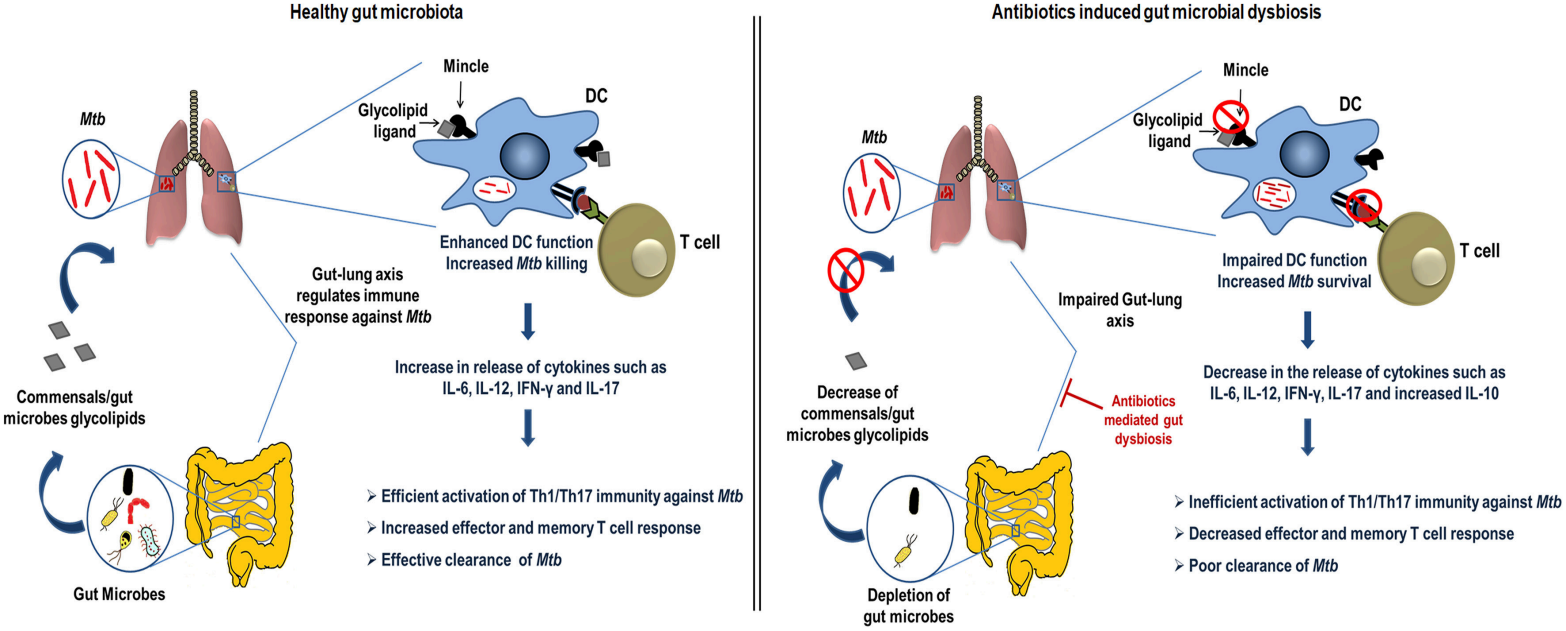
Conceptual model of the study. During homeostasis, a healthy gut through microbial products such as glycolipids (synthetic analog, TDB that binds mincle) regulates lung immune response during *Mtb* infection. Gut commensals bacteria derived glycolipids reach lung via blood stream. In *Mtb* infected lungs, they bind to the mincle receptor expressed on DCs leading to their activated phenotype and functions such as production of immunoregulatory cytokines (IL-6, IL-12), which in turn elicits the CD4 T cells differentiation to Th1 and Th17 cells via the release of IFN-γ and IL-17 cytokines, respectively. Further, there is generation of memory response and protective immunity against *Mtb* in lungs. This immunoregulation through gut microbiota is disturbed upon the Abx induced dysbiosis. Abx depletes the beneficial commensals population, which is responsible for the impairment of DCs function and hence the dysregulated lung anti-*Mtb* immunity.

In the current study, we demonstrated the crucial role of gut microbiota in regulating lung DCs function against *Mtb* via mincle receptor. These data provide insights into gut-lung axis and Abx induced dysregulation of lung immunity. Abx induced gut dysbiosis caused hyporesponsiveness of lung DCs, leading to attenuated T cell response in TB, highlighting important implications of broad-spectrum antibiotic use (Figure 10).

Gut microbiota/ derived products influence host immunity by binding to PRRs expressed on immune cells. Intestinal microbes have been implicated in the pathogenesis of many diseases such as rheumatoid arthritis and type 1 diabetes through innate immune pathways (39, 40). For instance, nucleotide-binding oligomerization domain-containing protein 1 (NOD-1) receptor has been reported to recognize circulating microbiota-derived peptidoglycan, influencing the function of bone marrow derived neutrophils (41). Moreover, protection in experimental autoimmune encephalomyelitis (EAE) model was shown to be mediated via polysaccharide A from commensal Bacteroides fragilis (42). We have recently shown that gut microbial disturbances rendered the mice more susceptible to TB (2). Other studies have suggested changes in gut microbiota composition upon *Mtb* exposure (1, 43). However, there is scarcity of findings that sufficiently depicted the interaction of gut and lung in shaping the host immune response against *Mtb*.

Mincle is copiously expressed by DCs (44). DCs are sentinels of the immune system and the only antigen presenting cells (APCs) that can independently activate and differentiate naïve T cells to various CD4 T cell subsets (27). Initially, we observed that gut dysbiosis downregulates the mincle expression in lung and is associated with the compromised anti-*Mtb* immunity. This corresponds well with the diminished mincle levels on lung DCs and their compromised immune response against *Mtb*.

This data have direct relevance to host anti-*Mtb* immune response, linking the aberrant T cell response upon Abx treatment. Gut dysbiosis expanded the *Mtb* specific Tregs. Tregs are known to inhibit the Th1 cells by secreting TGF-β or IL-10 via cognate interaction (45). The fact that Th17 and Tregs are known to reciprocally regulate each other (46), justifies the observed decrease in Th1 and Th17 response upon gut dysbiosis. Another factor that dampens the host immunity during *Mtb* infection is the presence of exhausted T cells (35). However, mincle stimulation reduced the pool of *Mtb* specific Tregs and exhausted T cells in microbiota disrupted animals.

Here, we propose that the interaction between gut and lung during TB is mediated through lung myeloid DCs; that upon gut dysbiosis displays impairment in antigen presentation, activation and ability to stimulate CD4 T cells. Recently, signals delivered by gut microbes were documented to influence functions of innate immune cells (14, 47). DCs are critical innate cells involved in restricting *Mtb* survival and activating T cell response (26, 48). Abx treatment reduced the mincle expression on lung myeloid CD11c^+^CD11b^+^ DCs and impaired the functional responsiveness of DCs, consistent with the attenuated effector and memory CD4 T cells response in lung.

Nonetheless, mincle activation via TDB administration in dysbiotic mice successfully restored the DCs phenotype and function such as antigen presentation, activation, cytokines secretion such as IL-12 and IL-6, phagocytosis and *Mtb* restricting ability. Further, blockade of mincle activation on DCs with anti–mincle antibody was shown to dampen their *Mtb* killing activity. This suggests the importance of gut microbiota in modulating the lung DCs through mincle.

Furthermore, microbiota disrupted animals had changes in their gut microbiota profile, which may be correlated to the observed restoration of immune-defects in the lung upon mincle stimulation. More specifically, we found increase in beneficial commensals such as *Lactobacillus* and *Bacteroides*. Members of *Lactobacillus* genus are known to induce Th1 immunity (49). Further, *Bacteroides* constitutes a consortium of numerous commensals that are responsible for major fermenting processes, glycolipids production and promoting systemic Th1 immune response (50). These dramatic shifts of gut microbes toward beneficial microbial consortium can be related well with the observed protective Th1 immune response against *Mtb*.

Interestingly, we found that *Oscillospira* was decreased in antibiotics treated mice infected with *Mtb*. Mincle stimulation in these mice resulted in *Oscillospira* levels similar to control animals. It has been shown that *Oscillospira* abundance is directly correlated with intestinal permeability, thus mediating the translocation of gut bacteria derived products from the gut to blood circulation (51). Additionally, systemic activation via translocation of gut microbial products from the intestine into the blood circulation has been well-documented (18). Thus, we believe that increased intestinal permeability contributes to release of gut-derived products that influence lung immunological response. Gut microbial products such as glycolipids may release into the bloodstream and reach lungs. These glycolipids might bind mincle receptor expressed on the lung DC and trigger them to further induce activation of CD4 T cells and thus restricts *Mtb*.

Administration of *Lactobacillus plantarum*, which was found to be diminished upon Abx treatment and reported to possess glycolipids that signal through mincle receptor (22), elevated the expression of mincle and MHC-II on lung DCs along with restored anti-*Mtb* immunity in lungs. This study suggests that Abx-associated immune defects involve depletion of gut microbial population/ derived products that modulate mincle activation in lung DCs during *Mtb* infection. Although the exact mechanism through which *Lactobacillus* modulated the lung immunity still needs to be further studied. Nonetheless, we showed that both the Abx-associated lung DCs and T cell dysfunction were rescued by TDB and *Lactobacillus* supplementation, although limitations include the precise identification of gut microbial products and their targets *in vivo*.

Overall, the gut microbiota is indispensable for maintaining DC-mediated lung immune homeostasis through mincle. Abx can disrupt these innate regulatory pathways in the lung and induce immune dysfunction. This has a detrimental impact on both innate and adaptive immunity against *Mtb* infection. It will be an interesting line of future investigation to study the therapeutic role of various glycolipids released by gut commensals in restricting *Mtb* survival. Further, understanding of host-commensals interaction may open avenues for the development of new approaches to manipulate gut flora in order to maintain and enhance beneficial gut commensals. Commensals possessing glycolipids that can signal through mincle may have therapeutic potential to be used as probiotic or in adjunctive therapy to boost host immunity in TB.

## Ethics Statement

All experiments and protocols were in accordance with the Institutional Animal Ethics Committee of IMTECH and accredited by Committee for the Purpose of Control and Supervision of Experiments on Animals (No. 55/1999/ CPCSEA), Govt. of India.

## Author Contributions

JA and SN conceived the idea and designed work. SN, SP, and HB performed the experiments. Analysis, data interpretation and manuscript writing was done by JA and SN.

### Conflict of Interest Statement

The authors declare that the research was conducted in the absence of any commercial or financial relationships that could be construed as a potential conflict of interest.
