# Supplementary material for: Gut Microbiota Regulates Mincle Mediated Activation of Lung Dendritic Cells to Protect Against Mycobacterium tuberculosis
Source: Front Immunol. 2019 May 28;10:1142. doi: 10.3389/fimmu.2019.01142 (PMC6558411; doi:10.3389/fimmu.2019.01142)
Supplement: Supplementary file 1 [file Data_Sheet_1.doc]

**Gut microbiota regulates mincle mediated activation of lung dendritic cells to protect against *Mycobacterium tuberculosis***

**Shikha Negi^1^, Susanta Pahari^1,3^, Hilal Bashir^1^ , Javed N Agrewala^1, 2*^**

^1^ Immunology Division, CSIR-Institute of Microbial Technology, Chandigarh, India

^2^ Indian Institute of Technology, Ropar-140001, India

^3^ Immunology Division, Texas Biomedical Research Institute, San Antonio, Texas, USA.

* Correspondence: Javed N Agrewala, Email address: jagrewala@gmail.com

**Supplementary figure legends**

**Figure S1.**


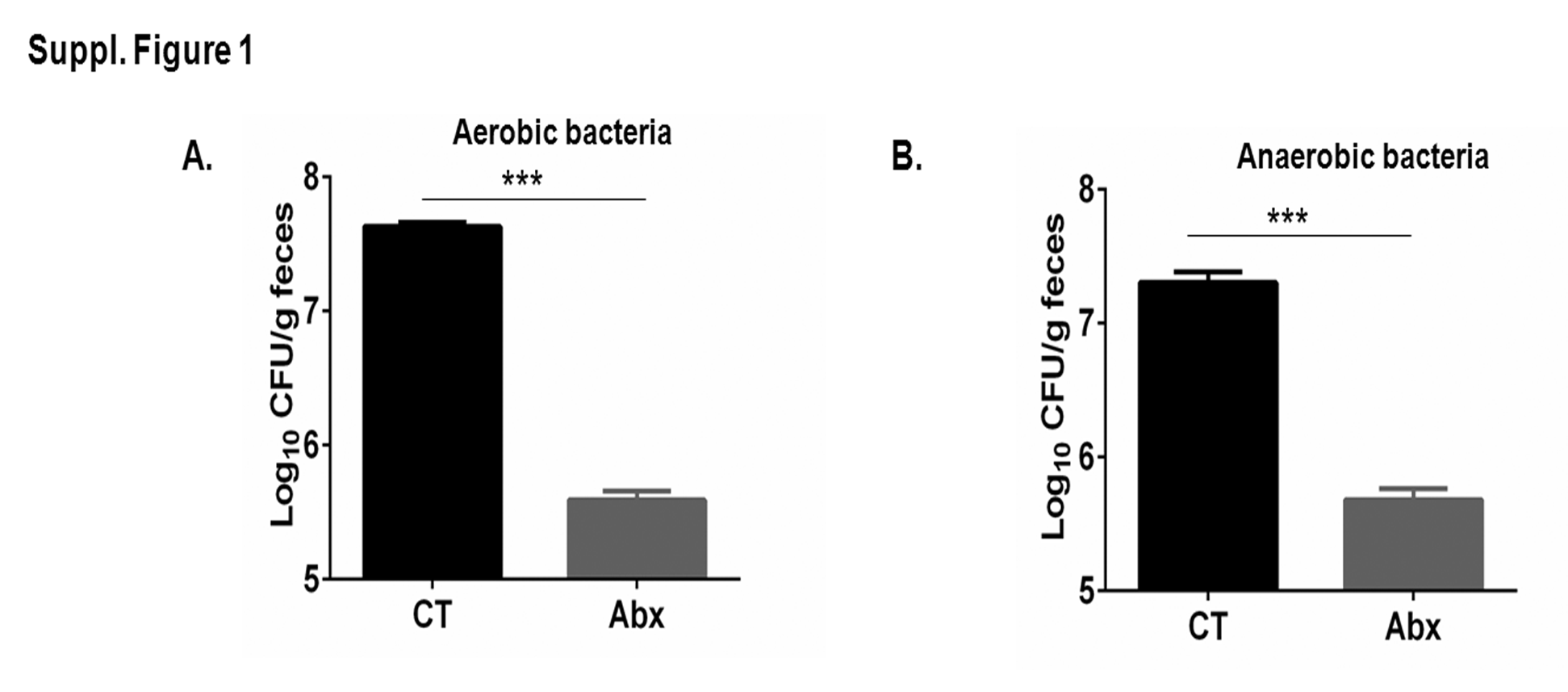


**Figure S1.** Abx treatment disrupts the gut microbial population. Mice were treated with broad-spectrum Abx cocktail (Vancomycin, Neomycin, Metronidazole). After 15 d, fecal samples were assessed for the cultivable (A) Aerobic and (B) Anaerobic bacteria by CFU assay. Data expressed as mean ± SD of two independent experiment (n=5 mice/group). ***p<0.001. CT: Control mice not treated with Abx; Abx: mice treated with Abx.

**Figure S2.**


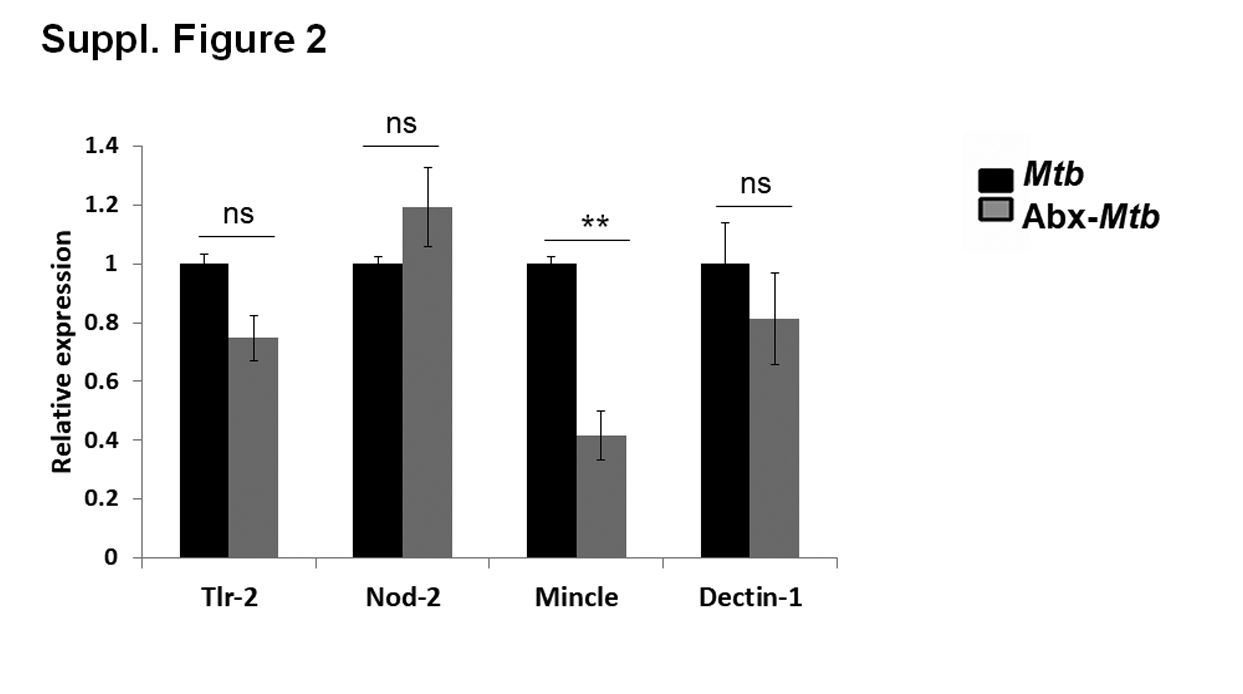


**Figure S2.** Mice were given Abx in drinking water for 4 wk followed by *Mtb* infection (~100 CFU) through aerosol challenge. After 4 wk, lung cells were isolated and examined for the expression of innate receptors such as TLR-2, NOD-2, Mincle and Dectin-1 through qRT-PCR. Bar graphs depict fold change normalized to β-actin as reference gene. ns: non-significant, **p<0.01. Data are from two independent experiments (n=5-6 mice/group). *Mtb*: *Mtb* challenged mice; Abx-*Mtb*: mice treated with Abx prior to *Mtb* infection.


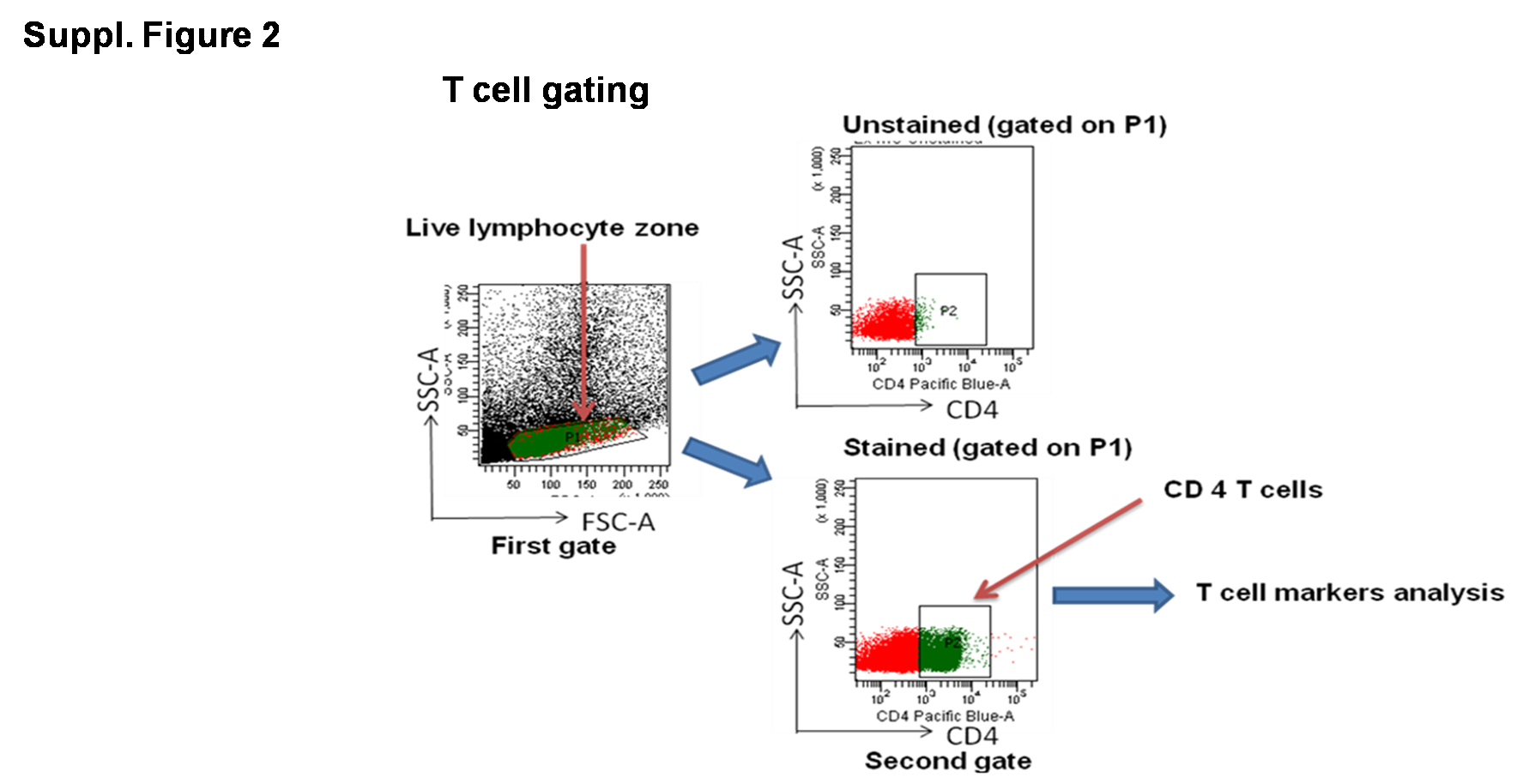


**Figure S3.**

**Figure S3.** Gating strategy for CD4 T cells analysis. Cells were initially gated on the live lymphocyte zone and then, second gate on SSC-A and CD4 T cells. Further, T cell markers analysis was done on these gated cells only.

**Figure S4.**


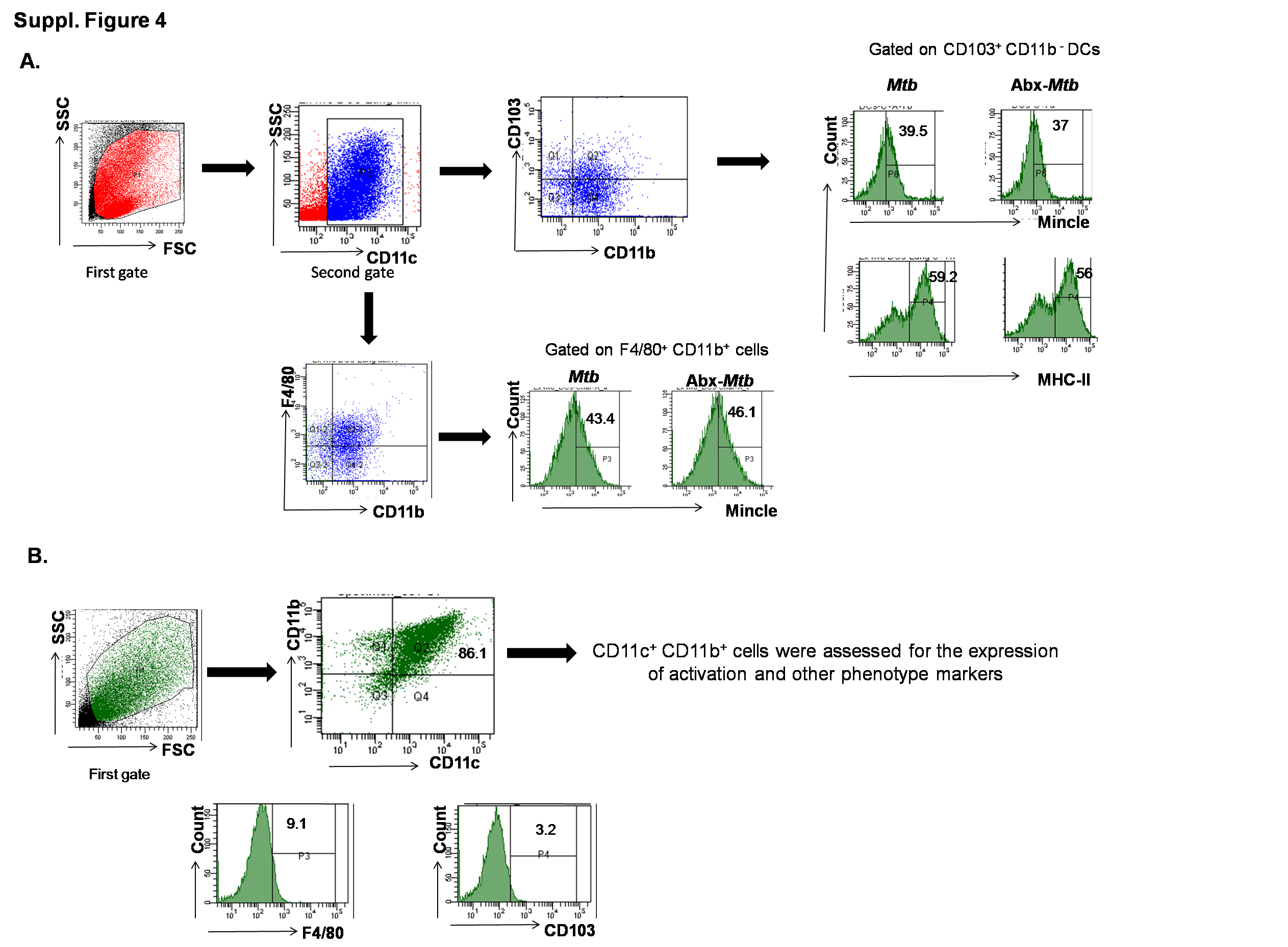


**Figure S4.** (A) Flow cytometry representation of lung cell suspensions. First, the live cell population was gated on the basis of FSC/SSC. Cells in first gate were further gated as CD11c-positive cells and on these CD103 *vs* CD11b and F4/80 *vs* CD11b dot plot were made. Further, the mincle expression was examined on the CD103^+^ CD11b^-^ DCs (classical DC1) and F4/80^+^ CD11b^+^ cells (activated macrophages) which did not show any change upon Abx treatment. *Mtb*: *Mtb* challenged mice; Abx-*Mtb*: mice treated with Abx prior to *Mtb* infection. (B) Lung cells were sorted and enriched for DCs (CD11c^+^ CD11b^+^ population) as explained in methods. Further, for flow cytometry analysis, cells were gated on live cells and thereafter, analyzed for the expression of CD11c *vs* CD11b which constitute >86% of population. Other cells positive for CD103 (3.2%) and F4/80 (9.1%) constitute a small subset of cells.

**Figure S5.**

**
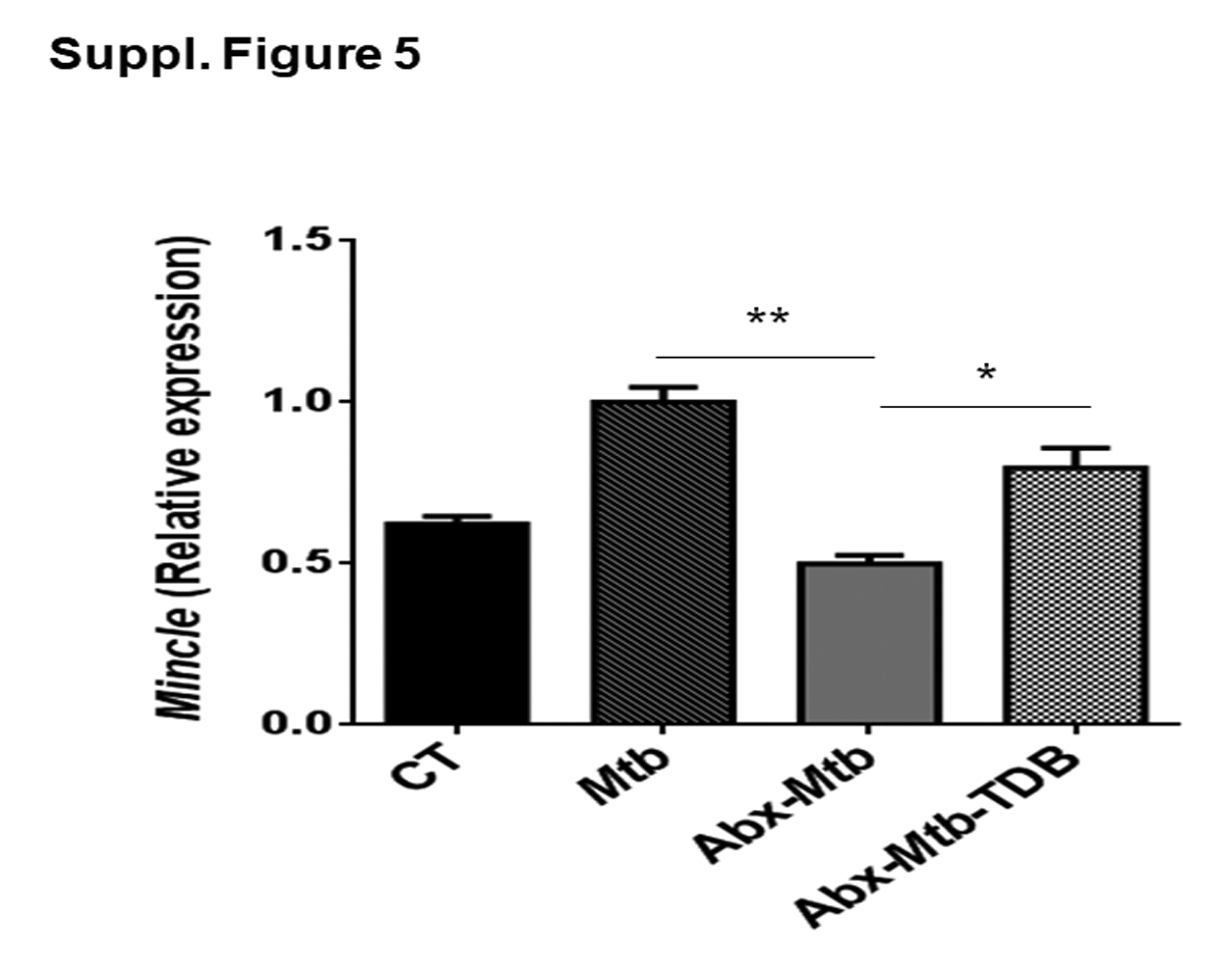
**

**Figure S5.**  Mice were treated with Abx treated prior to aerosol challenge with *Mtb* (~100 CFU) and oral administration of TDB (50 µg) as mentioned in legend to Fig.1. After 3 wk, lung cells were sorted for CD11c^+^ DC population and assessed for the expression of mincle by qRT-PCR. Bar graphs depict fold change normalized to β-actin (reference gene). **p<0.01,*p<0.05. Data are from two independent experiments (n=5-6 mice/group). CT: control mice without Abx treatment; *Mtb*: *Mtb* challenged mice; Abx-*Mtb*: mice treated with Abx prior to *Mtb* infection; Abx-*Mtb*-TDB: mice with disrupted gut microbiota prior to *Mtb* infection and TDB administration.

**Figure S6.**

**
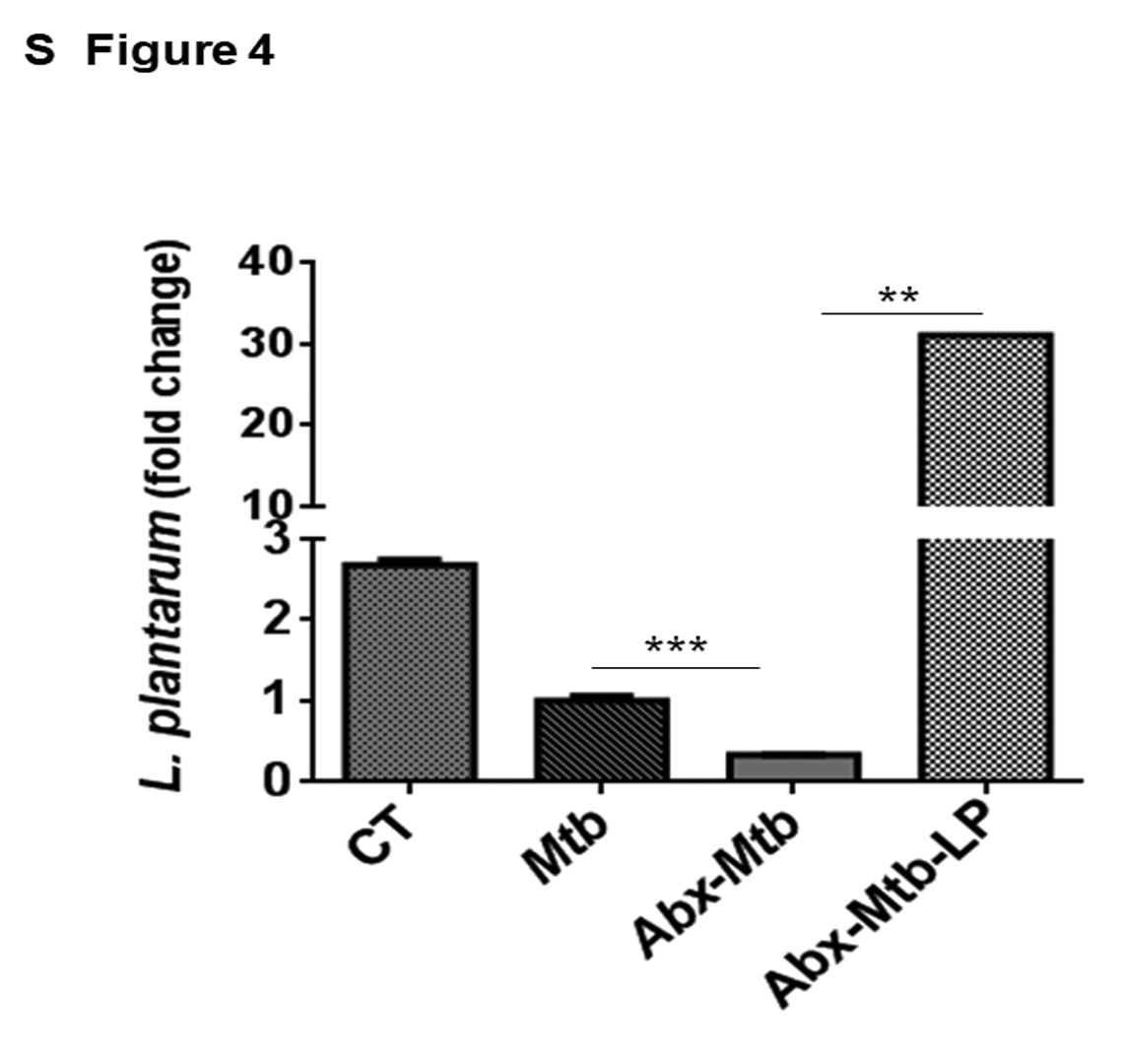
**

**Figure S6. *Lactobacillus plantarum* was decreased in *Mtb* infected mice with gut dysbiosis.** Mice were given Abx in drinking water for 4 wk followed by *L. plantarum* (10^8^ CFU per mice) administration every other day for 2 wk prior to *Mtb* infection until sacrifice. After 4 wk, fecal DNA was isolated and subjected to qPCR analysis. Bar graph depicts the abundance of *Lactobacillus plantarum*. Abundance depicted as fold change normalized with a universal bacterial primer, **p<0.01, ***p<0.001. Data are from two independent experiments (n=5-6 mice/group).

**Figure S7.**


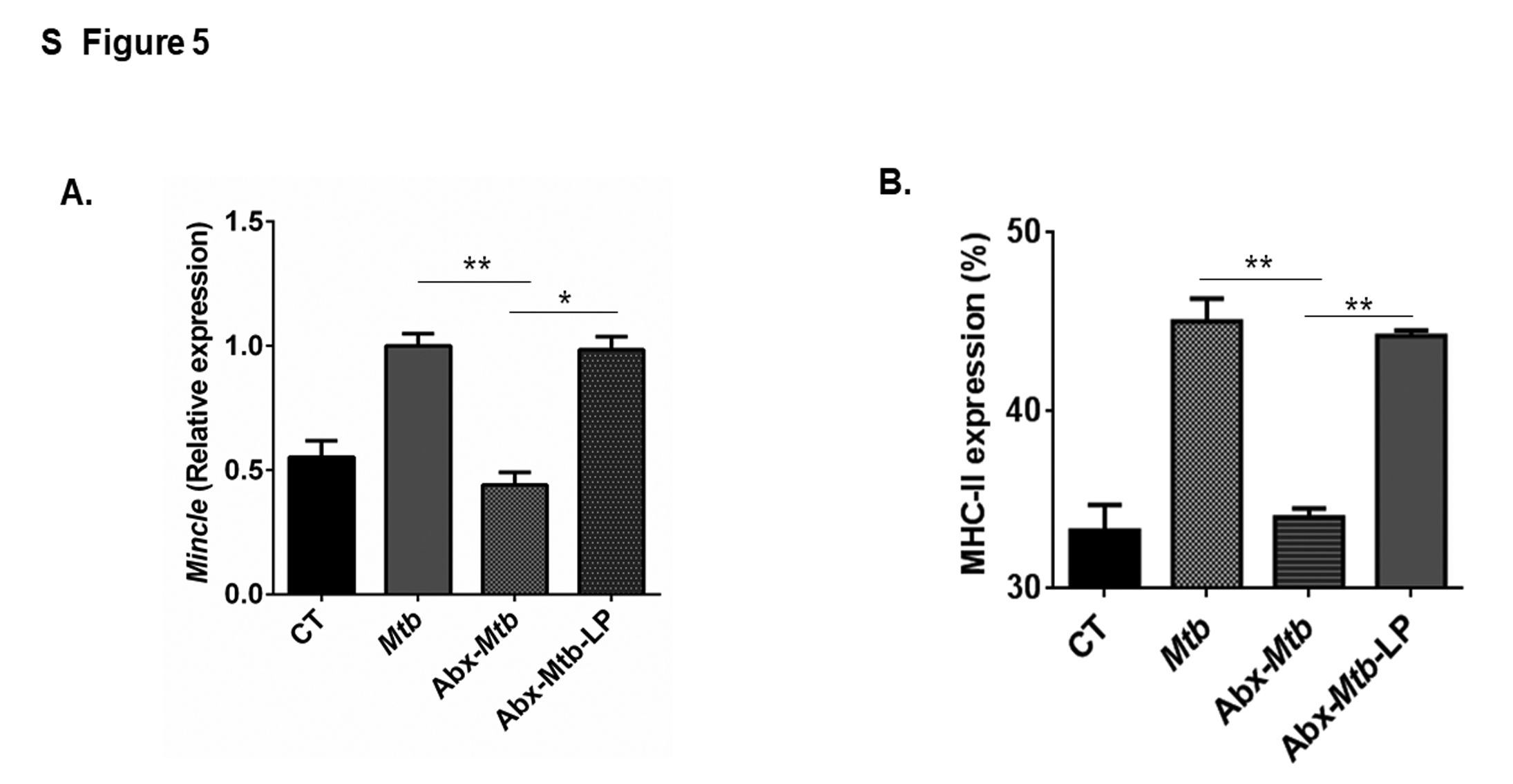


**Figure S7. Administration of *Lactobacillus plantarum* enhances the expression of mincle and MHC-II on lung DCs from gut-disrupted mice infected with *Mtb*.** Mice were given Abx in drinking water for 4 wk followed by *L. plantarum* (10^8^ CFU per mice) administration every other day for 2 wk prior to *Mtb* infection until sacrifice. After 4 wk, lung cells were sorted for CD11c^+^ DC population and assessed for the expression of (A) mincle by qRT-PCR, depicted as fold change in bar graph normalized to β-actin and (B) MHC-II *via* flow cytometry, bar graph represent the percentage of cells gated on CD11c^+^ CD11b^+^ population. *p<0.05, **p<0.01. Data are from two independent experiments (n=5-6 mice/group).

**Supplementary Table 1.** PCR Primer sequences used in the study.

| Primer | Sequence |
| --- | --- |
| *Mincle* | Fw: 5'-TGCTACAGTGAGGCATCAGG-3'  Rv: 5'-GGTTTTGTGCGAAAAAGGAA-3' |
| *Tlr-2* | Fw: 5'-AAGAGGAAGCCCAAGAAAGC-3'  Rv: 5'-CGATGGAATCGATGATGTTG-3' |
| *Nod-2* | Fw: 5'-CTTCATTTGGCTCATCCGTAG-3'  Rv: 5'-CTGGAGATGTTGCAGTACAAAG-3' |
| *Dectin-1* | Fw: 5'-AATCCTGTGCTTTGTGGTAG-3'  Rv: 5'-GACTGAGAAAAACCTCCTGTAG-3' |
| *Bacteriodes* | Fw: 5'-GAGAGGAAGGTCCCCCAC-3'  Rv: 5'-CGCTACTTGGCTGGTTCAG-3' |
| *Lactobacillus* | Fw: 5'-AGCAGTAGGGAATCTTCCA-3'  Rv: 5'-CACCGCTACACATGGAG-3' |
| *Enterococcus* | Fw:5'-CCCTTATTGTTAGTTGCCATCATT-3'  Rv: 5'-ACTCGTTGTACTTCCCATTGT-3' |
| *Lactobacillus plantarum* | Fw: 5'-TGGATCACCTCCTTTCTAAGGAAT -3'  Rv: 5'-TGTTCTCGGTTTCATTATGAAAAAATA-3' |
| Total bacteria | Fw: 5'-ACTCCTACGGGAGGCAGCAGT-3'  Rv: 5'-ATTACCGCGGCTGCTGGC-3' |
| *β-actin* | Fw: 5'-AGAGGGAAATCGTGCGTGAC-3'  Rv: 5'-CAATAGTGATGACCTGGCCGT-3' |
| *GAPDH* | Fw: 5′-TCGCTCCTGGAAGATGGTGATGG -3  Rv: 5′-GGCAAATTCAACGGCACAGTCAAG-3′ |
